# Supplementary material for: Computational analysis of the metal selectivity of matrix metalloproteinase 8
Source: PLoS One. 2020 Dec 4;15(12):e0243321. doi: 10.1371/journal.pone.0243321 (PMC7717551; doi:10.1371/journal.pone.0243321)
Supplement: S1 Table — (DOCX) [file pone.0243321.s002.docx]

**S1 Table.** **Relative distance between the water oxygen atom and the carbonyl carbon atom.**

| **Structure** | **Distance (Å)** |
| --- | --- |
| **WT MMP8 Zn(II)** | 2.848 |
| **WT MMP8 Cu(II)** | 2.779 |
| **WT MMP8 Mg(II)** | 2.881 |
| **WT MMP8 Co(II)** | 2.814 |
| **H197Q MMP8 Zn(II)** | 2.920 |
| **H197Q MMP8 Cu(II)** | 2.808 |
