# Supplementary material for: Computational analysis of the metal selectivity of matrix metalloproteinase 8
Source: PLoS One. 2020 Dec 4;15(12):e0243321. doi: 10.1371/journal.pone.0243321 (PMC7717551; doi:10.1371/journal.pone.0243321)
Supplement: S2 Table — (DOCX) [file pone.0243321.s003.docx]

**S2 Table.** **Torsion angles in the simulated ligand for the i position where peptide bond gets cleaved by MMP8**.

| **Structure** | **φ (degree)** | **ψ (degree)** | **ω (degree)** |
| --- | --- | --- | --- |
| **Collagen III ^1^** | -16.873 | 118.121 | 177.076 |
| **WT MMP8 Zn(II)** | -26.334 | 163.721 | 176.002 |
| **WT MMP8 Cu(II)** | -41.326 | 169.894 | 177.520 |
| **WT MMP8 Mg(II)** | -20.584 | 166.216 | 175.837 |
| **WT MMP8 Co(II)** | -29.333 | 173.367 | 177.840 |
| **H197Q MMP8 Zn(II)** | -32.251 | 164.460 | 176.217 |
| **H197Q MMP8 Cu(II)** | -33.635 | 166.833 | 178.636 |

**Reference**

1. Boudko SP, Engel J, Okuyama K, Mizuno K, Bachinger HP, Schumacher MA. Crystal structure of human type III collagen Gly991-Gly1032 cystine knot-containing peptide shows both 7/2 and 10/3 triple helical symmetries. J Biol Chem. 2008;283(47):32580-9.
